# Altered intrinsic functional network connectivity is associated with impulsivity and emotion dysregulation in drug-naïve young patients with borderline personality disorder

**DOI:** 10.1186/s40479-023-00227-y

**Published:** 2023-06-19

**Authors:** Wanyi Cao, Ying Liu, Mingtian Zhong, Haiyan Liao, Sainan Cai, Jun Chu, Shuxin Zheng, Changlian Tan, Jinyao Yi

**Affiliations:** 1 Medical Psychological Center, The Second Xiangya Hospital, Central South University, Changsha, 410011 Hunan China; 2 Medical Psychological Institute, Central South University, Changsha, China; 3 National Clinical Research Center for Mental Disorders, Changsha, Hunan China; 4 Center for Studies of Psychological Application, School of Psychology, South China Normal University, Guangzhou, China; 5 Department of Radiology, The Second Xiangya Hospital, Central South University, Changsha, Hunan China

**Keywords:** Borderline personality disorder, Impulsivity, Emotion dysregulation, Default mode network, Central executive network, Salience network

## Abstract

**Background:**

Despite impulse control and emotion regulation being altered in borderline personality disorder (BPD), the specific mechanism of these clinical features remains unclear. This study investigated the functional connectivity (FC) abnormalities within- and between- default mode network (DMN), salience network (SN), and central executive network (CEN) in BPD, and examined the association between aberrant FC and clinical features. We aimed to explore whether the abnormal large-scale networks underlie the pathophysiology of impulsivity and emotion dysregulation in BPD.

**Methods:**

Forty-one young, drug-naïve patients with BPD (24.98 ± 3.12 years, 20 males) and 42 healthy controls (HCs; 24.74 ± 1.29 years, 17 males) were included in resting-state functional magnetic resonance imaging analyses. Independent component analysis was performed to extract subnetworks of the DMN, CEN, and SN. Additionally, partial correlation was performed to explore the association between brain imaging variables and clinical features in BPD.

**Results:**

Compared with HCs, BPD showed significant decreased intra-network FC of right medial prefrontal cortex in the anterior DMN and of right angular gyrus in the right CEN. Intra-network FC of right angular gyrus in the anterior DMN was significantly negatively correlated with attention impulsivity in BPD. The patients also showed decreased inter-network FC between the posterior DMN and left CEN, which was significantly negatively correlated with emotion dysregulation.

**Conclusion:**

These findings suggest that impaired intra-network FC may underlie the neurophysiological mechanism of impulsivity, and abnormal inter-network FC may elucidate the neurophysiological mechanism of emotion dysregulation in BPD.

## Introduction

Borderline personality disorder (BPD) is a severe mental disorder characterized by poor impulse control, dysfunctional emotion regulation, distorted self-image and instability in interpersonal relationships, and non-suicidal self-injury behavior [1]. Impulsivity in BPD can lead to various dangerous behaviors such as risky driving, substance abuse, aggression, self-harm and suicidality [2, 3]. Emotion dysregulation in BPD manifests as mood volatility and difficulty in controlling anger, which may lead to intense relationships and increased suicidal tendencies [4, 5]. However, the pathophysiology mechanisms of impulsivity and emotion dysregulation in BPD remain unclear.

Resting-state functional magnetic resonance imaging (fMRI) studies have found that impulsivity and emotion dysregulation in BPD were associated with functional abnormalities in the frontal cortex and precuneus [6, 7], anterior cingulate cortex [8], and insula [9]. Task-related brain imaging studies have also found that impulsivity and emotion dysregulation in BPD were associated with dysfunction in the prefrontal cortex [10, 11], anterior cingulate cortex [12], parietal lobes [13], amygdala and hippocampus [14]. However, some studies have suggested that the neural dysfunctions of BPD might occur at large-scale brain networks rather than in an independent brain region [15, 16]. Brain networks provide new insights into understanding the pathophysiological mechanisms of impulse control and emotion regulation in BPD [17–19]. Menon et al. proposed a triple network model (default mode network, DMN; salience network, SN; central executive network, CEN) to understand the neural physiopathology of affective, cognitive, and social functions in multiple psychiatric disorders, including BPD, major depressive disorder, bipolar disorder, and others [20]. The DMN, a task-negative network, is linked to social and affective cognitions and self-introspections [21]. The DMN can be divided into two major subdivisions: anterior DMN (aDMN, mainly involving the medial prefrontal cortex, mPFC) and posterior DMN (pDMN, mainly including the posterior cingulate cortex and precuneus) [22]. The SN, covering anterior and posterior parts of the insula and the anterior cingulate cortex, plays a critical role in information filtering, detection, and integration [23]. Lastly, the CEN, including left and right lateralized frontal-parietal regions, is associated with executive control processes and the cognitive processes during goal-directed behaviors [24].

Neuroimaging studies in BPD have found intra-network dysfunctions in these three neural networks underlying impulsivity and emotion dysregulation with mixed results. Some studies found increased functional connectivity (FC) in the DMN was related to impulsivity [19, 25], while others revealed decreased FC in the DMN was associated with emotion dysregulation [16, 26]. Meanwhile, studies also found abnormal inter-network FC among the triple networks underlying emotion deficits, such as decreased inter-network FC between the CEN and SN as well as between the DMN and SN reflecting the instability of emotion regulation and emotion processing [18, 27]. However, there were several limitations in these studies. First, a seed-based method was used in a previous study [27]. One study has reported that large-scale networks are composed of several sub-networks [28], while the seed-based inter-region analysis has a limited capacity to explore the FC of sub-networks. Second, comorbidities may lead to biased results, while BPD samples in previous studies were comorbid with other psychiatric disorders (e.g., MDD, attention deficit hyperactivity disorder, and substance abuse) [16, 18, 26]. Third, previous studies in BPD did not eliminate the potential medication effects [16, 18, 19], which might influence brain activation and FC [29, 30]. Finally, most studies paid attention to the association between intra-network or inter-network FC and clinical characters, while no study systematically explored whether the large-scale networks of intra- as well as inter- network FC in the three networks underlie the pathophysiology of impulsivity and emotion dysregulation in BPD.

In this study, we applied data-driven independent component analysis (ICA) to investigate FC within and between the DMN, CEN, and SN among drug-naïve BPD patients without comorbidities. Furthermore, we assessed the relationship between FC in triple networks and clinical characteristics (e.g., impulse control and emotion regulation) in BPD. We aimed to explore whether abnormal large-scale networks underlie the pathophysiology of clinical features in BPD. Understanding pathophysiological mechanisms behind BPD will provide insights for accurate diagnosis and targeted treatment. Based on previous findings, our hypotheses were as follows: (a) BPD may show abnormal intra- and inter- network functional connectivity; (b) abnormal intra- and inter- network functional connectivity may be associated with specific clinical features in BPD.

## Methods

### Participants

This study was approved by the ethics committee of Second Xiangya Hospital. All participants provided signed informed consent forms.

We recruited young patients with BPD from the outpatients of the Second Xiangya Hospital, Central South University. We also recruited healthy controls (HCs) from the surrounding community through advertisements.

The diagnosis of BPD was conducted by two experienced psychiatrists based on the Structured Clinical Interview for Axis II disorders (SCID-II) of the Diagnostic and Statistical Manual of Mental Disorders, Fourth Edition (DSM-IV). Each participant also received a structured clinical interview to exclude past or current Axis I psychiatric disorders (e.g., schizophrenia, attention deficit hyperactivity disorder, post-traumatic stress disorder, and substance abuse disorders) [31]. Participants with neurodevelopmental disorders, physical disorders of known psychiatric consequences (e.g., seizure disorder, hypothyroidism, brain injury) and contraindications to MRI were also excluded.

To control the effect of medication and comorbidities, we used a BPD subsample without medication and without comorbidities in this study. Among the 101 participants with BPD (28 males and 73 females) recruited, we excluded 20 patients with medication, 9 patients comorbid with major depressive disorder and 2 patients comorbid with bipolar disorder. There were 70 BPD patients left (21 male and 49 female). Additionally, we matched the number of male and female patients by randomly selecting half of the female sample (25 women) to control the effect of gender on results. Therefore, the BPD sample used in this study was a subsample of the patient pool we had.

Two qualified psychiatrists interviewed HCs using the SCID-I/II. Control participants were excluded if they met the criteria for contraindications to MRI, past or current history of any DSM-IV Axis I or Axis II disorder, current medical problem, or history of psychiatric disorders among first-degree relatives. Finally, forty-five age- and gender- matched HCs (18 males and 27 females) participated in this study.

### Psychometric instruments

#### Barratt Impulsiveness Scale-11th version (BIS-11)

The BIS-11 was used to measure impulsivity level [32], which includes 30 items. Each item is scored on a 4-point Likert scale, from 1 (never) to 4 (always). The BIS-11 consists of three factors: non-planning impulsivity (11 items), motor impulsivity (11 items) and attentional impulsivity (8 items). The total score of BIS-11 ranges from 30 to 120, with higher scores indicating greater impulsivity. The Chinese version of the BIS-11 showed good reliability and validity [33]. In this study, the Cronbach's *α* of BIS-11 was 0.81.

#### Cognitive Emotion Regulation Questionnaire (CERQ)

The 36-item CERQ was used to assess cognitive emotion regulation strategies when encountering negative events [34], which includes nine types of specific strategies (a 5-point Likert scale, range 1–5). Self-blame, rumination, blaming others, and catastrophizing are regarded as maladaptive subscale (CERQ-M), while putting into perspective, acceptance, refocus on planning, positive reappraisal, and positive refocusing are regarded as adaptive subscale. In the current study, only the CERQ-M was used to assess negative emotion regulation strategies, with scores ranging from 16 to 80. The Chinese version of CERQ has shown acceptable reliability and validity [35]. In this study, the Cronbach's *α* of the CERQ-M subscale was 0.85.

#### Center for Epidemiological Studies Depression Scale (CES-D)

The 20-item CES-D was used to evaluate participants’ depressive symptoms [36]. Each item of CES-D is scored on a 4-point Likert scale from 1 (never) to 4 (very often), and the total score of CES-D ranges from 20 to 80. The CES-D has shown adequate psychometric properties in Chinese population [37]. In the current study, the CES-D had excellent internal consistency (Cronbach's *α* = 0.89).

#### State-Trait Anxiety Inventory (STAI)

The STAI is a self-reported anxiety questionnaire, including state anxiety inventory (SAI) and trait anxiety inventory (TAI) subscales [38]. Each subscale contains 20 items rated on a 4-point Likert scale from 1 (never) to 4 (always). The total score of each subscale is from 20 to 80. The Chinese version of the STAI has shown good reliability and validity [39]. The SAI (Cronbach's *α* = 0.93) and TAI (Cronbach's *α* = 0.90) subscale had good reliability in this study.

### Functional magnetic resonance imaging data acquisition

MRI was performed with a 3.0 T Philips Ingenia scanner. The participants were instructed to lie on their backs with their eyes closed, to avoid systematic thinking, and to stay awake. Ear plugs and foam pads were used to reduce noise and head motion. Structural T1-weighted images were acquired with a three-dimensional 3D spoiled gradient recalled sequence (repetition time (TR) = 7.44 ms, echo time (TE) = 3.46 ms, slice thickness = 1.2 mm, field of view (FOV) = 240 mm, flip angle = 8°, matrix size = 240 × 240, voxel size = 0.60 × 1.0 × 1.0 mm^3^, slices = 301). Resting state fMRI sensitive to BOLD signal changes were obtained using a 6 min and 40 s gradient-echo echo-planar imaging sequence (TR = 2000 ms, TE = 30 ms, FOV = 240 mm × 240 mm, flip angle = 90°, matrix size = 128 × 128, slice thickness = 4 mm, slice spacing = 4 mm, voxel size = 1.88 × 1.88 × 4.0 mm^3^, slices = 36, volumes = 200).

Data preprocessing was performed in the software of Data Processing Assistant for Resting-State fMRI (DPARSF, http://www.restfmri.net/forum/DPARSF) [40], which is based on Statistical Parametric Mapping (SPM12, http://www.fil.ion.ucl.ac.uk/spm) and Resting-state fMRI Data Analysis Toolkit (REST, http://www.restfmri.net) [41]. We discard the first 10 volumes to minimize the initial instability of machine and the participants’ adaptation. The images were corrected for slices timing differences and realigned to correct head motions. After motion correction, the functional scans were normalized to the Montreal Neurological Institute (MNI) template with the T1 image, resampled to 3 × 3 × 3 mm^3^ voxel size, and smoothed with an 8 mm full-width half maximum Gaussian kernel. High resolution T1-weighted image was used to exclude structural abnormalities for each participant, and was segmented into gray matter, white matter, and cerebrospinal fluid to obtain whole-brain gray matter volumes. Previous research found that gray matter volume might influence brain functional activation [42]. Therefore, the gray matter volume of each participant was included as a nuisance covariate in data analysis to control the influence of differences in brain volume. Meanwhile, five BPD (1 male and 4 females) and three HC (1 male and 2 females) participants who had head translation greater than 2.0 mm or head rotation greater than 2.0° in any direction were excluded.

### ICA and selection of network-of-interest

A spatial ICA for all 83 participants was performed by using the Group ICA for fMRI toolbox (http://icatb.sourceforge.net). The preprocessed data was decomposed into 75 independent components using principal components analysis (PCA). The number of components was affirmed based on previous studies [28, 43], which proposed that high-model-order ICA models can refine components corresponding to known anatomical and functional segmentations and provide a more detailed and robust decomposition of subnetworks. Twenty ICAs (ICASSO, implemented in GIFT software) were performed to ensure the stability of the decomposition. This procedure resulted in a set of average group components that were back-constructed into single-subject space via group ICA-3 algorithm based on the compression and projection of PCA [44]. Mean of all quality index (Iq) was then used to assess the overall stability of the ICA decomposition. The mean Iq was 0.96 in this study, indicating a stable ICA decomposition. For each component, the spatial map of z-scores and its corresponding time course for each participant as well as the average z-map and time course were then obtained.

In terms of independent component selection, multiple spatial correlation analyses were conducted on 75 independent components average z-maps according to previously established templates [28]. Specifically, Allen and colleagues decomposed resting-state fMRI data of 603 participants into 75 independent components using a group-ICA framework in the GIFT software [28]. In this study, the SN, CEN and DMN were chosen as templates, and then spatial correlation analyses were performed between our 75 average z-maps of the independent components and these templates. The top three correlations between ICA component and network templates were as follows: anterior DMN (component 8, *r* = 0.463; component 38, *r* = 0.423; component 12, *r* = 0.268), posterior DMN (component 73, *r* = 0.552; component 56, *r* = 0.447; component 35, *r* = 0.309), SN (component 20, *r* = 0.536; component 60, *r* = 0.364; component 62, *r* = 0.274,), left CEN (component 49, *r* = 0.643; component 35, *r* = 0.200; component 60, *r* = 0.197), and right CEN (component 37,* r* = 0.661; component 53, *r* = 0.384; component 20, *r* = 0.171). Components that had the highest correlation coefficients with templates (anterior DMN, component 8; posterior DMN, component 73; SN, component 20; left CNN, component 49; right CNN, component 37) were then selected to represent the five networks. Therefore, 5 independent components were selected from all participants.

### Outcome measures

We extracted 5 independent components for each participant, each component’ z-map and its corresponding time course indexed the intra-network FC. Before calculating the inter-network FC, we further linearly detrended, despiked, and temporally filtered the time courses of all network-of-interest [45]. We computed Pearson's correlation coefficients between the time course of each pair of networks in the SN, DMN and CEN subsystems, and then transformed the coefficients into z-scores via Fisher's z-transformation in each participant. The transformed z-scores represented the inter-network FC of each pair of networks.

### Statistical analysis

We performed two-sample* t*-tests and chi-squared tests to compare the differences in demographic and clinical features between BPD and HC groups in SPSS 25 (SPSS Inc., Chicago, IL, United States), with Cohen’s *d* to reflect the effect size of group differences [46].

To analyze group effects of intra-network FC, we performed one-sample* t*-tests to compare the participants’ reconstructed spatial maps for each network and each group (*p* < 0.05, corrected for family-wise error correction, FWE). With a conjunction map of the one-sample *t*-test image as a network mask, we then applied two-sample *t*-tests to analyze participants' spatial z-maps, which included gender, age, education and gray matter volume as covariates. Also, we ran the sensitivity analyses with CES-D, SAI, TAI scores, gender, age, education and gray matter volume as covariates to explore whether the findings were valid and reliable. Results were corrected via a cluster-level *p*_*FWE*_ < 0.05 with a voxel-level threshold *p* < 0.001.

To detect group differences of inter-network FC, we transformed Pearson's correlation coefficients of each pair network into z values via Fisher r-to-z transformation. Then, we used two-sample* t*-tests to compare the z-scores between BPD and HC groups (*p* < 0.01 with Bonferroni correction for 10 pairwise correlations).

For the BPD group, we conducted partial correlation analyses (gender, age, education, and gray matter volume as covariates) to explore the association between clinical features (BIS-11 and CERQ-M subscales) and the z-scores that differed significantly between BPD and HC groups.

## Results

### Demographic and clinical variables

The final analysis included 41 patients with BPD (20 males and 21 females) and 42 HCs (17 males and 25 females). Table 1 summarizes demographic and clinical characteristics for each group. There were no significant group differences in age, sex, education, and gray matter volume. The patients with BPD scored significantly higher on BIS-11, CERQ-M, CES-D, SAI and TAI than HC participants.

**Table 1:** Demographic and clinical characteristics of the BPD and HC (Mean ± SD)

	BPD	HC	*t*/*χ*^*2*^	*p*	|Cohen’s *d*|
	*N* = 41	*N* = 42			
Age (years)	24.98 ± 3.12	24.74 ± 1.29	0.46	0.65	0.10
Sex (male: female)	20:21	17:25	0.58	0.45	0.17
Education (years)	15.90 ± 0.37	16.12 ± 0.67	-1.81	0.07	0.41
GMV (ml)	713.22 ± 197.95	769.35 ± 263.43	-1.10	0.28	0.24
BIS-11 scores					
Total	67.37 ± 8.53 (54 ~ 87)	59.51 ± 7.05 (45 ~ 78)	4.58	< 0.001	1.00
Attention	17.73 ± 3.43 (12 ~ 27)	14.51 ± 2.68 (9 ~ 20)	4.77	< 0.001	1.05
Motor	22.93 ± 3.49 (17 ~ 33)	21 ± 3.03 (15 ~ 28)	2.69	0.009	0.59
Nonplanning	26.71 ± 4.52 (20 ~ 40)	24 ± 3.41 (18 ~ 34)	3.08	0.003	0.68
CERQ-M	40.17 ± 5.71 (29 ~ 53)	34.24 ± 5.40 (25 ~ 43)	4.87	< 0.001	1.07
CES-D	35.73 ± 6.63 (26 ~ 53)	27.79 ± 4.91 (20 ~ 41)	6.22	< 0.001	1.36
SAI	37.41 ± 9.07 (22 ~ 62)	30.00 ± 5.76 (20 ~ 52)	4.46	< 0.001	0.98
TAI	41.90 ± 8.01 (26 ~ 58)	33.57 ± 5.00 (24 ~ 53)	5.70	< 0.001	1.25

*BPD* borderline personality disorder, *HC* healthy control, *|Cohen’s d|* absolute value of Cohen’ s *d*, *GMV* gray matter volume, *CERQ-M* maladaptive subscale of Cognitive Emotion Regulation Questionnaire, *BIS-11* the Barratt Impulsiveness Scale-11^th^ version, *CES-D* Center for Epidemiologic Studies Depression Scale, *SAI* State Anxiety Inventory, *TAI* Trait Anxiety Inventory; (): range of scores

### Identification of network-of-interest

**Fig. 1:**
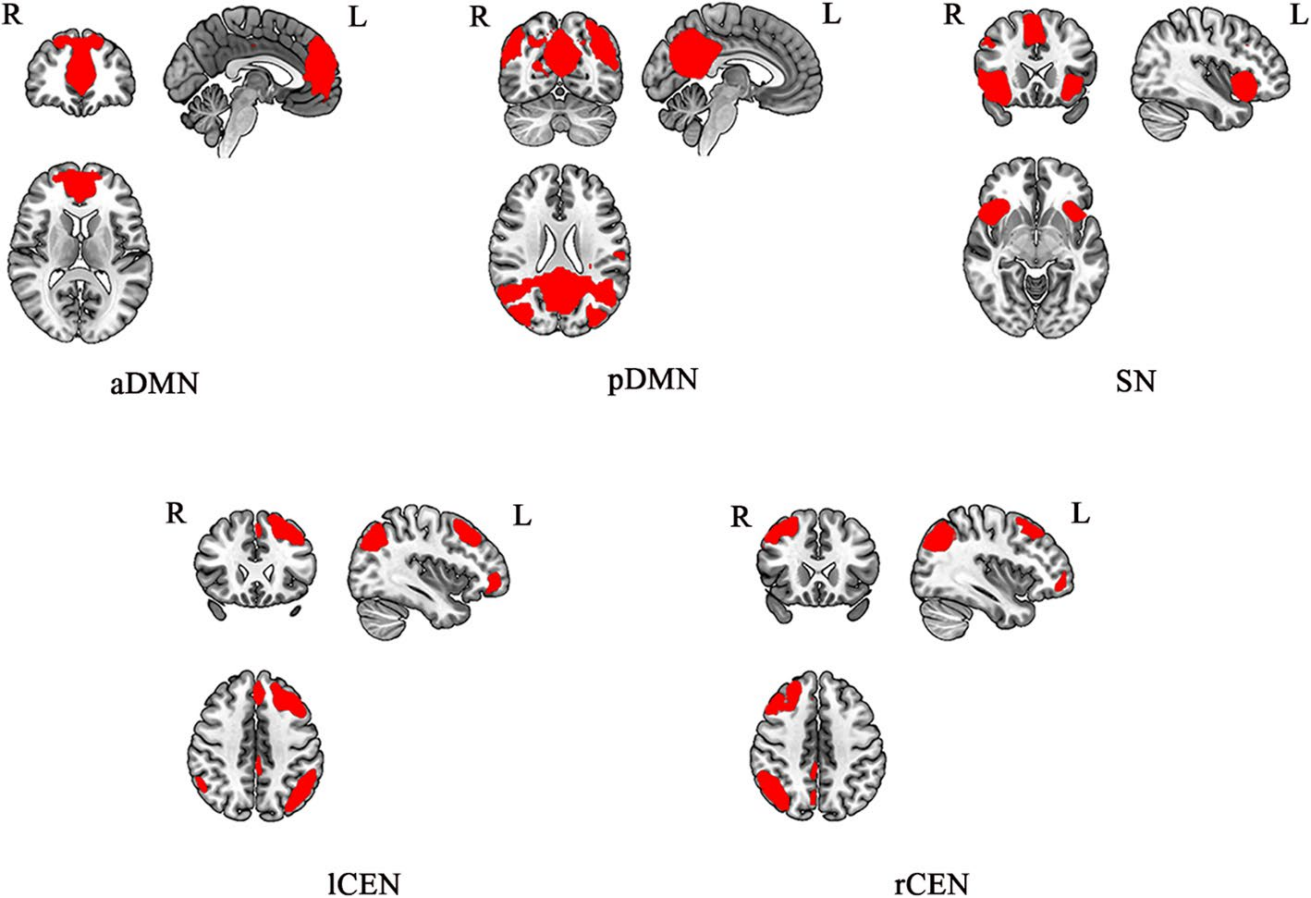
Spatial patterns of the DMN, CEN, and SN. aDMN, anterior default mode network; pDMN, posterior default mode network; SN, salience network;lCEN, left central executive network; rCEN, right central executive network. R, right; L, left

Figure 1 shows the combined spatial map of the network-of-interest for each group revealed by the one-sample *t*-tests (*p*_*FWE*_ < 0.05). The aDMN mainly consisted of the mPFC and anterior cingulate cortex, whereas the pDMN mainly involved the precuneus and posterior cingulate cortex. The rCEN mainly comprised of right dorsal lateral prefrontal cortex, right angular gyrus and the right inferior parietal lobule, whereas the lCEN mainly consisted of the left dorsal lateral prefrontal cortex, left supramarginal gyrus and left inferior parietal lobule. The SN mainly included the cingulate cortex and insula.

### Group differences in intra-network FC

**Fig. 2:**
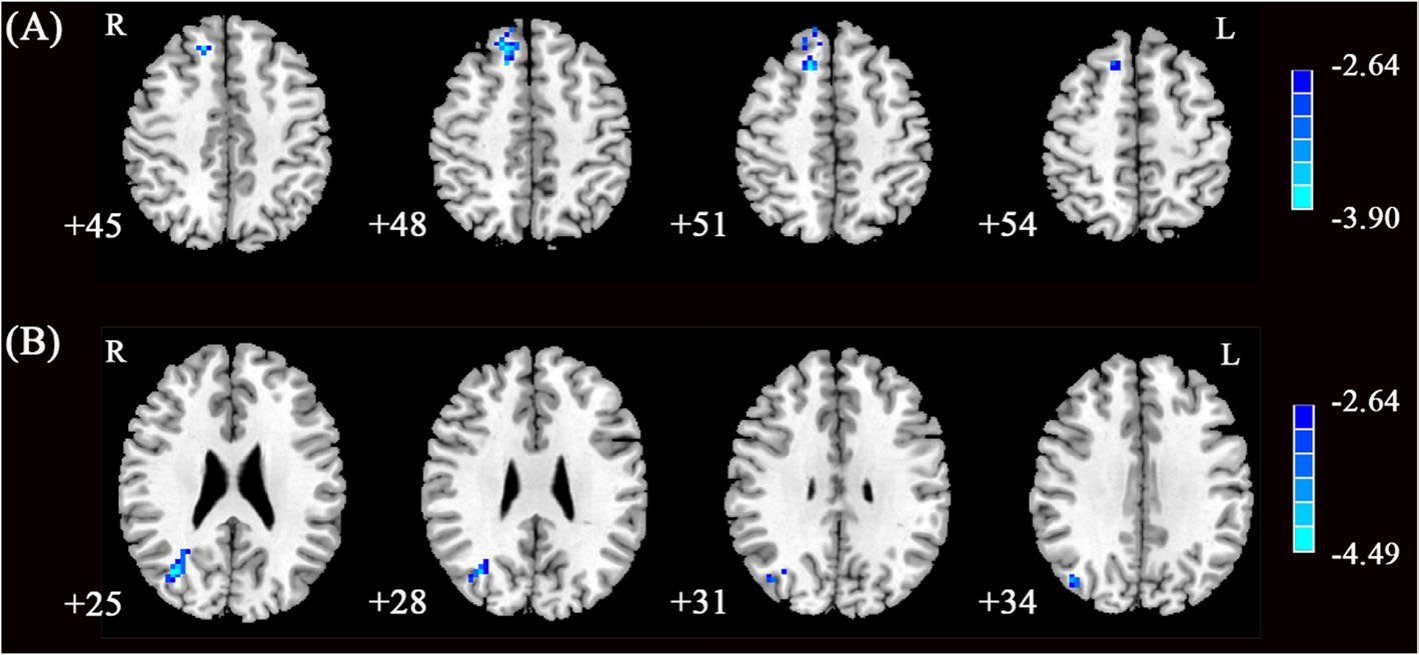
Group differences in intra-network FC. **A** Group differences in intra-network FC of right mPFC (x = 15, y = 21, z = 51; voxel = 38) in the anterior DMN. **B** Group differences in intra-network FC of right angular gyrus (x = 33, y = -69, z = 24; voxel = 48) in the right CEN. Cool colors indicating decreased intra-network FC in BPD patients compared with HCs. R, right; L, left

Compared with HCs, the BPD group had significantly decreased mean FC in aDMN (HC: 1.05 ± 0.17; BPD: 0.94 ± 0.17; *p* = 0.004) and rCEN (HC: 0.99 ± 0.14; BPD: 0.89 ± 0.13; *p* = 0.002), while there were no significant group differences in pDMN, lCEN, and SN. The comparisons of network z-maps revealed that BPD had significantly decreased intra-network FC of the right mPFC in the aDMN, and of the right angular gyrus in the rCEN than HCs (Fig. 2), while there were no significant group differences in intra-network FC of the anterior cingulate gyrus in the aDMN and of the right dorsal lateral prefrontal cortex in rCEN. No significant group differences in intra-network FC in the pDMN, lCEN, and SN were detected. With the CES-D, SAI, TAI scores, gender, age, education and gray matter volume as covariates, the group differences in intra-network FC were consistent with the above results (Table 2).

**Table 2:** Group differences in intra-network FC between BPD and HC

Network region	Hemisphere	BA	voxel	MNI			*T*
				x	y	z	
aDMN							
mPFC	Right^a^	8	38	15	21	51	3.90
	Right^b^	8	37	15	27	48	4.61
rCEN							
angular gyrus	Right^a^	39	48	33	-69	24	4.49
	Right^b^	39	56	42	-75	33	4.41

^a^with gender, age, education as covariates; ^b^ with CES-D, SAI and TAI, gender, age, education as covariates

### Group differences in inter-network FC

Compared with the HC group (*r* = 0.18 ± 0.25), the BPD group (*r* = -0.01 ± 0.22) had significantly decreased inter-network FC between pDMN and lCEN (*p* < 0.001, Bonferroni corrected; Table 3). No other significant group differences in inter-network FC were found among aDMN, rCEN and SN (*p* > 0.05).

**Table 3:** Group differences in inter-network FC (*r*) between BPD and HC

Inter-network FC	BPD	HC	*t*	*p*	|Cohen’s *d*|
aDMN-pDMN	0.03 ± 0.27	0.20 ± 0.27	-2.880	0.005	0.63
aDMN-SN	0.05 ± 0.27	-0.02 ± 0.29	1.120	0.422	0.25
aDMN-lCEN	0.40 ± 0.26	0.46 ± 0.26	-1.096	0.421	0.23
aDMN-rCEN	0.24 ± 0.21	0.37 ± 0.21	-2.926	0.005	0.62
pDMN-SN	0.03 ± 0.21	-0.06 ± 0.25	1.723	0.105	0.39
pDMN-lCEN	-0.01 ± 0.22	0.18 ± 0.25	-3.882	< 0.001	0.81
pDMN-rCEN	0.31 ± 0.26	0.37 ± 0.27	-1.078	0.203	0.23
SN-lCEN	0.04 ± 0.24	0.01 ± 0.27	0.485	0.614	0.12
SN-rCEN	0.14 ± 0.19	0.08 ± 0.23	1.195	0.244	0.28
lCEN-rCEN	0.36 ± 0.24	0.51 ± 0.31	-2.462	0.012	0.54

*FC* functional connectivity, *BPD* borderline personality disorder, *HC* healthy control, *|Cohen’s d|* absolute value of Cohen’ s *d,* > 0.80: large effect size; aDMN: anterior default mode network, *pDMN* posterior default mode network, *SN* salience network, *lCEN* left central executive network, *rCEN* right central executive network; *p* < 0.001 (Bonferroni corrected)

### Partial correlations between FC and psychometric measures in BPD

Correlation results revealed that the intra-network FC of right angular gyrus in the rCEN was negatively correlated with BIS-attention (*r* = -0.34, *p* = 0.04) in BPD (Table 4); the intra-network FC of right mPFC in the aDMN was marginally negatively correlated with BIS-total (*r* = -0.30, *p* = 0.07), BIS-attention (*r* = -0.32, *p* = 0.05) in BPD (Table 4). The inter-network FC between pDMN and lCEN was negatively correlated with CERQ-M subscale scores (*r* = -0.40, *p* = 0.02) in BPD (Table 4). No other significant correlations between brain imaging variables and clinical characters were found in BPD.

**Table 4:** Correlations between altered FC values and clinical features in BPD

	mPFC		angular gyrus		pDMN-lCEN	
	*r*	*p*	*r*	*p*	*r*	*p*
BIS-total	-0.30	0.07	-0.23	0.18	0.16	0.34
BIS-attention	-0.32	0.05	-0.34	0.04*	0.25	0.14
BIS-motor	-0.14	0.39	-0.20	0.25	-0.05	0.79
BIS-Nonplanning	-0.22	0.20	-0.02	0.90	0.15	0.38
CERQ-M	0.24	0.15	0.01	0.57	-0.40	0.02*

*FC* functional connectivity, *BPD* borderline personality disorder, *mPFC* medial prefrontal cortex, *pDMN* posterior default mode network, *lCEN* left central executive network, *BIS* Barratt Impulsiveness Scale-11^th^ version, *CERQ-M* maladaptive subscale of Cognitive Emotion Regulation Questionnaire, *p < 0.05

## Discussion

This is the first study, to our knowledge, that investigated FC within and between the CEN, DMN, and SN in drug-naïve patients with BPD, and explored the relationship between impaired network connectivity and core symptoms of BPD. BPD had decreased intra-network FC of right mPFC in the aDMN as well as of right angular gyrus in the rCEN than HCs. In the BPD group, the decreased intra-network FC of right angular gyrus in the rCEN was significantly negatively correlated with attention impulsivity, and the decreased inter-network FC between pDMN and lCEN was significantly negatively correlated with emotion dysregulation. These findings highlighted that abnormal intra- and inter- network FC were associated with impulsivity and emotion dysregulation in BPD, respectively, which supported our hypotheses.

BPD showed decreased intra-network FC of right mPFC in the aDMN than HCs. The DMN is decomposed into two subsystems: the aDMN more involved in self-referential processing, and the pDMN more in episodic memory (Lee et al., 2020). Previous studies have found structural abnormalities of mPFC [47] and abnormal resting state FC of DMN [16] in BPD. Bechara proposed that the activity of the mPFC is influenced by top-down control mechanisms arising from the prefrontal cortex, and modulated by bottom-up emotional signals exerted by the amygdala [48]. The mPFC, linking control and emotion, plays an important role in cognitive control and affective processing [48]. Therefore, BPD with mPFC abnormalities might have various clinical problems, such as behavioral disinhibition, emotion problems, damaged social functioning [49–51]. The marginally significant correlations between right mPFC and BIS-total as well as BIS-attention in our study supported that dysfunction of right mPFC in the aDMN in BPD might underlie the deficit in impulse control.

In this study, BPD also had decreased intra-network FC of right angular gyrus in the rCEN. The angular gyrus is located at the back of the inferior parietal lobule [52]. Previous studies have reported decreased FC in inferior parietal lobule within the fronto-parietal network in BPD [19] and reduced gray matter volume in the inferior parietal lobule [53] in BPD. These functional and structural impairments of inferior parietal lobule in BPD implied that inferior parietal lobule is an important neuroimaging marker of the BPD. The inferior parietal lobule is involved in top-down attention and also associated with deficits in visuospatial processing in BPD [54, 55]. The CEN is crucial for cognitive control functions such as inhibitory control, complex decision making, and planning [20]. We found that the decreased intra-network FC of angular gyrus in the rCEN was significantly associated with higher attention impulsivity (referring to poor cognitive control and concentration), which might indicate the more severe the alteration of this network, the poorer the inhibitory control in BPD. Decreased intra-network FC of angular gyrus in the rCEN might be the neurophysiological mechanism of impulsivity in BPD.

Besides intra-network FC findings, we also observed decreased pDMN-lCEN inter-network FC alterations in BPD. Notably, the time courses between pDMN and lCEN correlated negatively in the BPD group but positively in the HC group. In a healthy brain, a positive correlation seems unexpected and inconsistent with the notion of anti-correlation between the DMN and CEN [20]. Based on high-model ICA experiments, Allen et al. confirmed that both DMN and CEN networks had functional subnetworks [28]. Using ICA method, Smith et al. also found that distinct subnetworks within the DMN had special connective patterns among themselves and with other functional networks [56]. The current results suggested that different subsystems of DMN and CEN might have specific inter-network FC patterns. The alteration in inter-network FC of pDMN-lCEN in BPD group might indicate impaired suppression mechanism between these two subnetworks. Moreover, we found a significant negative correlation between scores of CERQ-M subscale and inter-network FC of pDMN-lCEN in BPD. Emotional dysregulation is one of the main clinical characteristics and concerns of the clinical intervention in BPD [57]. The negative correlation between inter-network FC of pDMN-lCEN and affective dysregulation in BPD might provide a biological explanation for the emotion dysfunction in BPD. Therefore, altered inter-network FC between pDMN and lCEN might be crucial to clarify the emotion dysfunction of BPD.

However, we did not reveal abnormal intra- and inter- network FC in SN. As an important component in the triple network model, the SN is involved in filtering and detecting internal and external salient stimuli and plays an important role in monitoring the interactions between the DMN and CEN, acting as a “switching” [20]. The present result suggested that BPD might have normal switching function between task-negative and task-positive processing.

This study has several strengths. First, by excluding medication and comorbidities, we made the patients with BPD in this study highly homogenous, which helps us to better understand the neurobiological mechanism of BPD. Second, previous large-scale study have found significant gender differences in resting state FC [58], and we included ratio-matching female and male BPD to control the gender effect on results and overcome the limitation of using predominantly female samples in previous studies. Nevertheless, several potential limitations should be considered. First, we investigated the relationship between resting-state functional network and clinical features (impulsivity and emotion dysregulation) in BPD. Although the clinical features have been assessed by well-established self-reported scales and the spontaneous BOLD signals might reflect actual neuronal activity underlying human cognitive and emotion processing in the resting state, task fMRI related to impulse control and emotion regulation would be warranted to extend our findings in the future. Second, we primarily focused on a well-known triple-network model, but we did not include other networks related to cognitive function and emotion regulation in this study. Further study can explore more extensive brain networks, such as the limbic system. Third, the homogeneous patients might also limit the generalizability of our results to the broader BPD population, since BPD is often comorbid with other mental disorders. In the future, we should use different subtypes of BPD (e.g. patients with or without comorbidity) to examine whether our results could be replicated and serve as potential biomarkers of BPD. Fourth, we did not include clinical control group in this study, which limits conclusions about the specificity of the findings to BPD. In the future, it is necessary to include clinical control groups, such as bipolar disorder group. Fifth, as a cross-sectional study, this research cannot definitively illustrate the causal relationship between abnormal brain networks and clinical features in BPD. Therefore, longitudinal studies are necessary to capture the network biomarkers in BPD.

## Conclusion

In summary, dysfunctional connectivity in DMN and CEN subnetworks related to impulsivity, and aberrant inter-network connectivity between pDMN and lCEN was correlated with emotion dysregulation in BPD. These findings suggested that abnormal intra- and inter-large-scale networks might underlie the pathophysiology mechanism of impulsivity and emotion dysregulation in BPD. Targeting impulsivity and emotional regulation is critical for preventing impulsive behaviors and their consequences in patients with BPD, and these findings provide potential circuits that may be used in novel target approaches. One previous study found that the repetitive transcranial magnetic stimulation (rTMS) had significant effects on modulating impulsivity and emotion dysregulation in certain brain areas [59], and our results provided further support for the application of novel target approaches in BPD.

## Data Availability

The datasets used and/or analysed during the current study are available from the corresponding author on reasonable request.
